# A unified spatial multigraph analysis for public transport performance

**DOI:** 10.1038/s41598-020-65175-x

**Published:** 2020-06-12

**Authors:** Yaoli Wang, Di Zhu, Ganmin Yin, Zhou Huang, Yu Liu

**Affiliations:** 1 Institute of Remote Sensing and Geographical Information Systems, Peking University, Beijing, 100871 China; 2 Beijing Key Laboratory of Spatial Information Integration and Its Applications, Peking University, Beijing, 100871 China

**Keywords:** Environmental social sciences, Socioeconomic scenarios, Sustainability, Computational science

## Abstract

Public transport performance not only directly depicts the convenience of a city’s public transport, but also indirectly reflects urban dwellers’ life quality and urban attractiveness. Understanding why some regions are easier to get around by public transport helps to build a green transport friendly city. This paper initiates a new perspective and method to investigate how public transport network’s morphology contributes significantly to its performance. We investigate the significance of morphology from the perspective of graph classification – by extracting the typical local structures, called “motifs”, from the multi-modal public transport multigraph. A motif is seen as a certain connectivity pattern of different transport modes at a local scale. Combinations of various motifs decide the output of graph classification, particularly, public transport performance. We invent an innovative method to extract motifs on complex spatial multigraphs. The proposed method is adaptable to unify complex factors into one simple form for swift coding, and depends less on observational data to handle data unavailability. In the study area of Beijing, we validate that the measure can infer varied public transport efficiencies and access equalities of different regions. Some typical areas with undeveloped or unevenly distributed public transport are further discussed.

## Introduction

Urban development is confronted with critical challenges in contemporary society. With a growing population residing in urban areas, a sustainable and inclusive urban public transport (PT) system draws significant attention, since a good PT system eventually helps to alleviate social exclusion^1^ in the urbanized environment. Understanding PT performance is the foundation to build an appropriate PT network. PT performance, including the efficiency and access equality, directly or indirectly affects residents’ daily travel convenience, urban congestion, adoption of green travel modes, people’s subjective experience, job accessibility by PT, and impression of a city^2,3^.

PT performance is spatially heterogeneous. We experience that getting around by PT is easier in some areas than in some others. Even within the same area, the PT travel time to different spots is heterogeneous. This paper aims to understand the drive of such spatial heterogeneity. We hypothesize that morphology of the PT network contributes significantly to its performance heterogeneity and investigate the association from a new perspective – graph classification. There is a good justification why morphology should be studied: 1) morphology is an effective factor as both other studies have discovered^4–9^ and this paper demonstrates below, 2) it avoids an extensive amount of data and thus data unavailability, 3) it provides the foundation for a unified and swift coding as this study proposes.

Although evaluation on PT performance is not brand new, previous studies are not widely feasible since they have applied methods that require a significant amount of posterior observational data. Such methods are hindered by cold start when observational data is not available. Especially at the stage of pre-assessment before a system is rolled out, the lack of observation makes data-driven approaches infeasible. For example, some measures are particularly designed based on observations for posterior evaluation^10,11^; the methods from geography require detailed demographic and behavioural information to measure travel convenience^12–14^; probabilistic models are built particularly in a data-driven manner^15^ that not only consumes large size of data but cannot explicitly explain the influential factors. The basic point is that network morphology should be fully deployed if it is an effective indicator for PT performance. For a similar purpose to avoid data dependency, Mishra *et al*.^16^ proposed less observation-based measures. They defined several improved connectivity measures on node, link, and regional levels. There are a few differences between their work and ours that mark our contribution. First, their measures are still defined in pieces and uniquely for each level, while our measure has a unified and simple mathematical form as we will demonstrate below. Second, we are able to categorise the connectivity patterns into genres (i.e., *motifs*) as a benefit of the unified measure. Third, our measure is validated by being applied to infer real PT travel time efficiency and equality while Mishra *et al*. did not demonstrate how their indices are related to real traffic.

A significant contribution of this work is to extract motifs from PT multigraphs for its performance assessment, which provides a swift and unified encoding of heterogeneous network properties. This inherits the concept of graph classification, a process to predict the category of a graph by the combination of *motifs* – typical functional structures that either occur significantly more than random^17^, or function-wise contribute to the identification of a graph^18^. Since the difference of PT performance is hypothetically owed to the network’s substructures of different connectivity patterns of multiple transport modes (e.g., bus, subway, walk) and transfers, a *motif* is regarded as a representation of such typical substructures attributed to which the network is identified. For instance, the subway-subway transfer efficiency is distinct from subway-bus transfer; consequently different connectivity patterns in a local area lead to varied PT travel efficiencies in general. However, graph classification for PT multigraphs, as for many spatial multigraphs, has big challenges (more discussions in 2). Graph boosting is frequently applied for graph classification, a method that calculates subgraph (i.e., motifs) frequencies to build the vector representation of a graph^19,20^. In a traditional sense, enumerating motifs of spatial multigraphs is not only confronted with the combinatorial complexity, but also intrinsically impossible. The continuous numerical edge weight has to be discretized and prior knowledge of motifs has to be ready. Neither of the conditions are satisfied for PT multigraphs. This paper proposes a convolution-inspired method to tackle with the challenges as demonstrated below. Urban planners can apply this method to facilitate the pre-assessment of PT network performance by investigating the extracted motifs.

## Results

**Figure 1:**
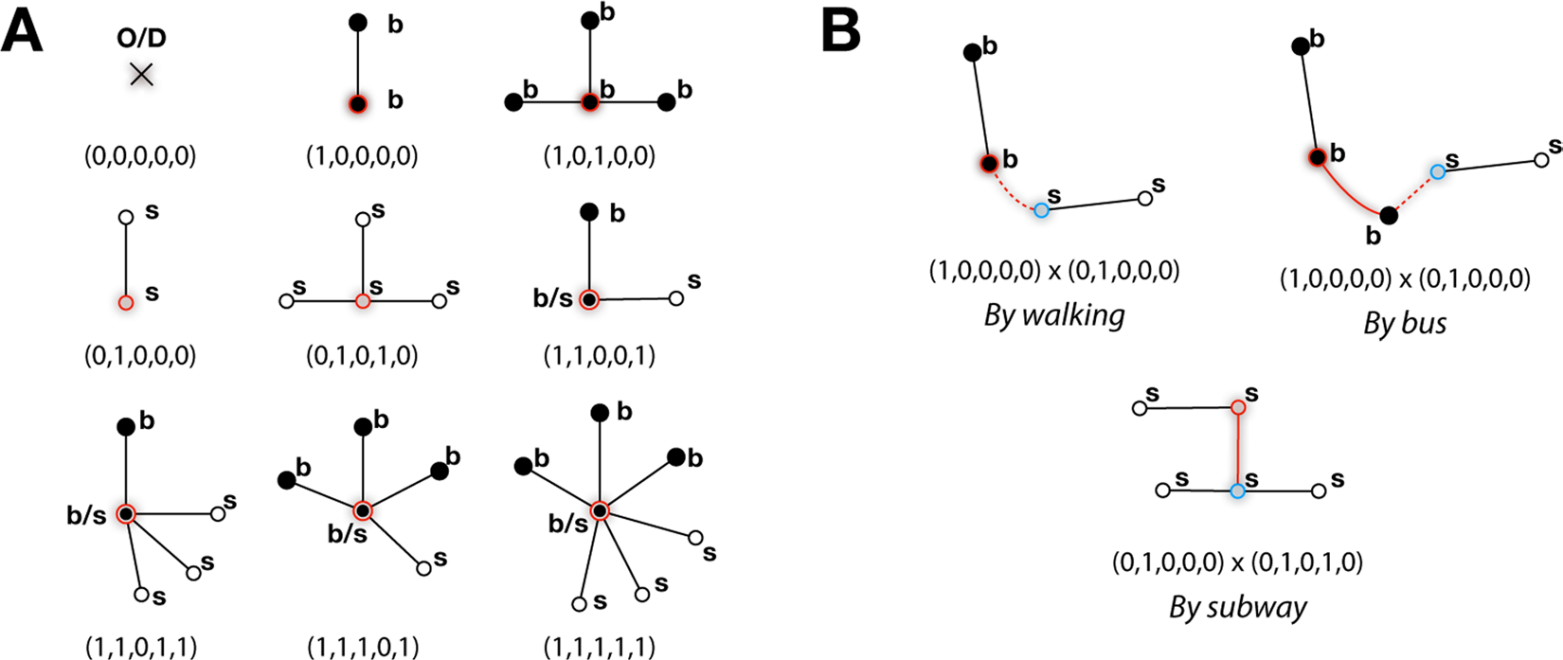
Illustration of some node types and connectivity patterns. (**A**) Some of the node types with the vector representations. The highlighted node with a shadow boundary is the ego node. A bus stop is a solid black dot marked with “b” and a subway station is a hollow dot with “s”. Depending on its connection with other nodes, the ego node can be (from top-left to bottom-right): a non-bus non-subway point (O/D), a bus non-transfer stop, a bus transfer stop, a subway non-transfer station, a subway transfer station, both a bus stop and a subway station (b/s), a bus stop and a subway transfer station, a bus transfer stop and a subway station, a bus transfer stop and a subway transfer station. The vector encodes different types of nodes. See *Methods* for details. (**B**) Some examples of the connectivities of different node types: a bus stop and a subway station connected by walking (dash red line), a bus stop and a subway station connected by bus (solid red line) and walking (dash red line), a subway non-transfer station and a subway transfer station connected by subway. The links are weighted by travel mode and distance with continuous numerical values, which makes it impossible to draw motifs in conventional sense.

A PT network is a multigraph – a similar concept as the *multilayer* network^21^ – because different travel modes can exist between the same pair of locations. The PT multigraph *M* in this study incorporates three basic networks: road, bus, and subway networks, of which different connectivity patterns yield varied functional performances. Note that for various cities transport modes can be diverse with other modes such as trams, shared bikes, and ferries. Detecting motifs in the PT spatial multigraph helps to understand what network substructures contribute positively or negatively to the PT performance. However, extracting *motifs* in a graph with *continuous numerical* edge weights is challenging because the amount of motifs can be infinite and there is a lack of prior knowledge on what is an effective motif. Consider the PT node types (A) and node connectivities (B) in Fig. 1 for an example. The combinatorial connectivity of different node types are theoretically infinite. One, the link weight ${w}_{ij}$ is continuously numerical, so two ostensibly similar local structures might be different just because of the link weights. Two, the number of neighbour nodes of an ego node is not capped, for which the connectivity patterns even without link weight can be infinite. It is thus difficult to define *motif* in the canonical sense (Supplementary Note 2).

### Convolution inspired motifs

We design a convolution inspired mechanism to handle the aforementioned difficulty. In the PT multigraph, a node represents a station/stop or an origin/destination (O/D) point that does not necessarily coincide with a station/stop. A link is a walk, bus route, or subway route connecting two locations. The convolution defined on the multigraph takes a simple mathematical form shown by Eq. (1), where ${v}_{i}$ is a node in a PT multigraph with $P({v}_{i})$ as the representation of node attributes (in the format of the 5-dimensional vector in Fig. 1), ${w}_{ij}$ is the edge weight considering travel speed and mode, and $t$ is the times of convolutions. Each dimension of the node vector corresponds to a particular function of the node. A benefit is that a uniform mathematical notation can distinguish nodes of different types. For example, as illustrated by Fig. 1A, a node can be a bus stop ((1, 0, 0, 0, 0), (1, 0, 1, 0, 0)) *or* a subway station ((0, 1, 0, 0, 0), (0, 1, 0, 1, 0)), be neither of them ((0, 0, 0, 0, 0), e.g, if an O/D), or be both of them (e.g., (1, 1, 0, 0, 1)) if the bus stop and subway station are sufficiently close. See *Methods* for more details.

1 $${P}^{(t)}({v}_{i})={P}^{(t-1)}({v}_{i})+\sum _{{v}_{j}\in N({v}_{i})}\,{w}_{ij}\times {P}^{(t-1)}({v}_{j})$$

As a solution to the aforementioned challenges, the logic of the proposed convolution is to convert a geometric problem into an algorithmic problem: instead of directly extracting motifs from a locality by applying structural isomorphism analysis, we vectorize nodes in the multigraph and calculate link weights with attributes; motifs are extracted as clusters of ego nodes’ vectors after aggregating the properties of directly and indirectly connected nodes via weighted links and connection topology. Every node in the multigraph is an ego node with regard to its neighbour nodes. Nodes are seen as *directly connected* if, in L-space, they are adjacent on the same PT line or are approachable by walk within the transfer threshold of 500 meters (see *Methods*, edge representation). Time of convolutions $t$ decides the range of nodes involved to calculate the output nodal vector of ego node.

The propagation is carefully designed to take into account the connectivity structure, distance, local travel modes, and transfers, all of which have been utilized to infer urban functions^22,23^. Intuitively, the assumption is that the linkage of varied travel modes and number of transfers matter for PT efficiency. For example, when looking at a segment of travel, if the segment needs to traverse two adjacent subway stations ${S}_{A}$ and ${S}_{B}$ (e.g., a link in Fig. 1), then the efficiency will be distinct from traversing the subway station ${S}_{A}$ and a directly linked bus stop. In a one-hop locality (i.e., $t=1$), a subway-subway connection is a different motif from subway-bus. When increasing the degree of propagation, the measure captures connectivity patterns of larger scale. Such encoding converts an areal connectivity structure to a nodal measure as the foundation of extracting motifs.

Based on the final nodal measure as a vector, we categorise the types of nodes by clustering the nodal measures with k-means and assigning each node with a label $\tau$. A continuous measure thus is turned into a categorical measure (*cluster*). Each cluster corresponds to a weakly defined *motif* that we hypothesize may hint at the PT network performance; certain connectivity patterns yield particular motifs which give rise to the network’s functional performance. We see at least two benefits of motifs: categorical measures are easier for human interpretation, and the patterns can be drawn flexibly with an arbitrarily selected number of clusters.

### Hints of motifs at observed PT performance: Case study

To validate the motifs and to understand the association between network’s structure and its performance, we examine how the motifs can infer PT performance, including PT efficiency and equality. The PT performance is estimated by mean and variance of observed PT travel time. The calculation is elaborated in *Observed PT performance* in *Methods*. We sample a few subareas modelled as multigraphs, each with several extracted motifs and a PT performance. The sample areas have a diameter of 4 kilometres to consider only short- to medium-distance travels. The reason to focus on short- to medium-distance travels is attributed to two interwoven reasons. First, PT for shorter distance travels significantly affects decision of travel mode because the last-mile problem prohibits the abandon of cars. The short distance PT travels hence deserve more attention. Second, the overhead costs before boarding and after alighting vehicles contribute to the diversity of travel efficiency^7^. The PT transfer and last mile distance to stations/stops that can be well captured by local PT multigraph structures are supposed to yield significant influence on local PT performance.

Graph classification is applied to associate motifs and PT performance as a mapping from measure-based domain to observation-based domain (see *Graph classification: from motifs to PT performance* in *Methods*). With the convolution-based measure, we conduct the case study within the 5^*th*^ Ring road of Beijing.

#### Detecting *motifs* on PT multigraph

**Figure 2:**
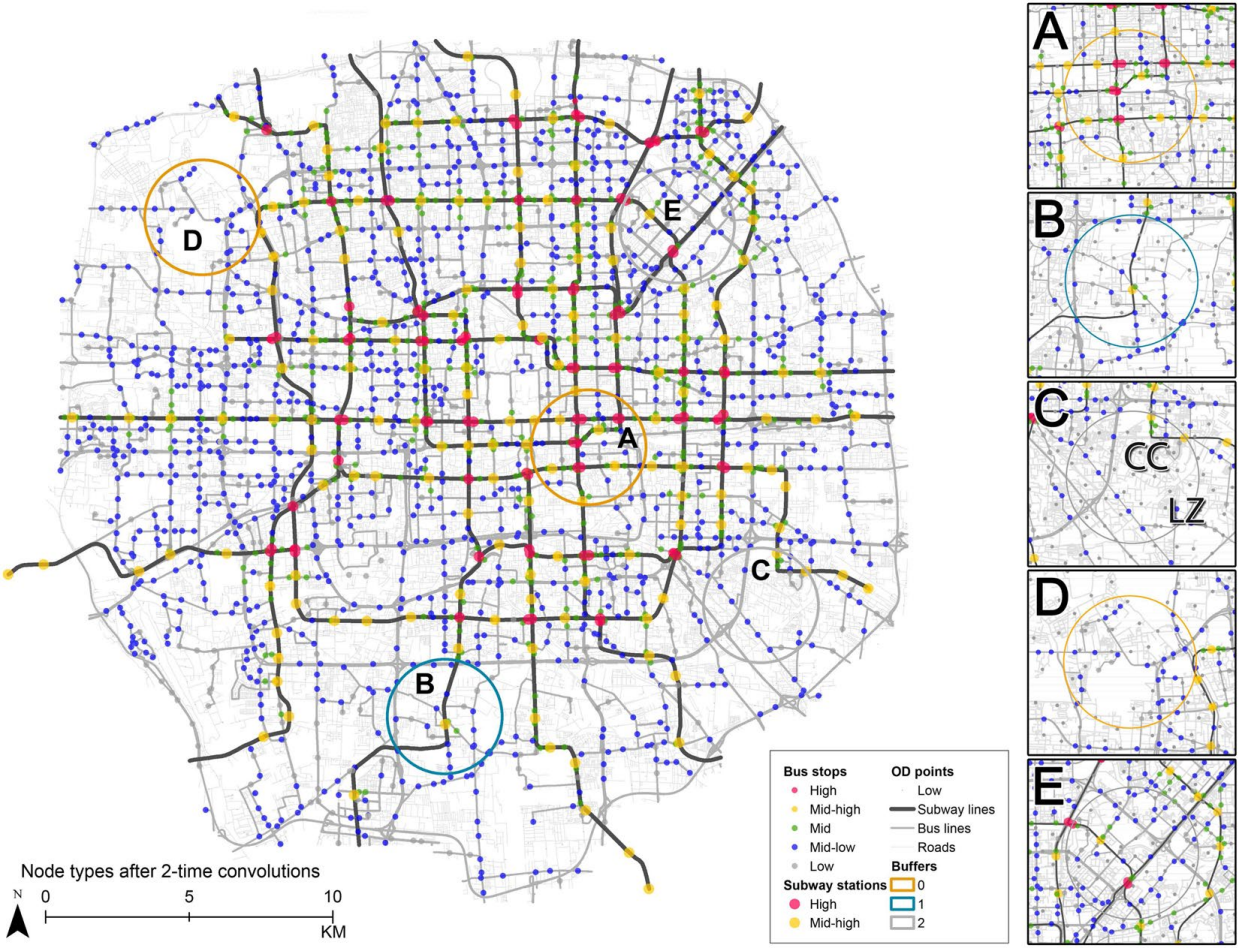
The distribution of five motifs (dot colours) after twice convolutions, and selected 2 km-radius subareas with travel time performance (circles coloured by ${l}_{\tilde{t}}$). Distributions of different motifs reflect the varied local PT system performances of subareas. The dot colour represents the accessibility of each node. The circle colour - for travel efficiency within a subarea - is different from the dot colour. The main figure demonstrates the whole distribution of motifs after convolutions. Each motif corresponds to a node’s function in its local context. In the legend “high” to “low” indicates its functional performance. Subfigures (**A**–**E**) are five sampled areas to demonstrate the association between motifs and the observed PT performance (designated by circle colour as different performance categories). The first three are typical patterns of each efficiency cluster whereas the rest two are unusual samples.

In the study area, the local connectivity yields 5 motifs after two convolutions, shown by the 5 colours in Fig. 2. With k-means the number of motifs is specified as input and can be adjusted accordingly. Each motif represents a tier of mobility, i.e., how easy to move around by PT if a traveller is at that node. By prior knowledge, subway stations with transfers have the highest mobility thanks to the benefit of transfer^4^ and the higher speed of subway, while an O/D point with a distance to any station/stop has the lowest mobility^24^; a bus stop, if closer to a subway station, should intuitively have higher mobility than one far from a subway station. The proposed measure is seen to catch the levels of mobility coinciding with the prior knowledge. When being clustered into 5 motifs, subway stations are distinguished into transfer (big red dots) and non-transfer stations (big yellow dots). Bus stops are clustered into a few tiers of mobility: the highest mobility (red) ones are close enough to subway transfer stations, the second highest (yellow) are near subway non-transfer stations, the next are the green ones, then blue ones, and finally grey ones. The measure helps to easily quantify the tiers of mobility and categorise the stops. Note that the number of mobility tiers is not fixed due to k-means. By designating more or fewer clusters, finer or coarser levels of mobility tiers can be fetched. The variables are also flexibly chosen to be built into the formula of node vector or edge weight.

#### Inferring PT performance with motifs

PT performance, including efficiency and equality, is a *region*-based measure calculated by the mean and variance of PT travel time $\tilde{t}$ of the sampled point pairs within the subarea (Supplementary Fig. 1). In the experiment we calculate both PT travel time and PT travel distance (see *The observed PT performance* in *Methods*). Hereafter we only stick to PT travel time $\tilde{t}$ and skip the analysis of distance $\tilde{d}$ since the analysis follows a similar manner. More discussions are given in Supplementary Note 6.

The *observed* PT travel time $\tilde{t}$ is clustered into 3 types considering both its mean and variance, of which the output is a target label ${l}_{\tilde{t}}$. The mean and the variance of $\tilde{t}$ respectively stand for the observed efficiency (or easiness) and the equality of PT travel. For visual clarity, we select 5 representative subareas to demonstrate the 3 types of ${l}_{\tilde{t}}$ (A–E in Fig. 2, the whole distribution is discussed in Supplementary Fig. 4). The orange ones have high mobility and high equality (high mean, low variance); the grey ones have lower efficiency but relatively high equality (low mean, low variance); the blue one is the worst with low efficiency and low equality (low mean, high variance).

Considering various orders of connectivity for nodes (different values of $t$ in Eq. 1) may yield slightly different results (Supplementary Note 5). This may be attributed to not only the size but also the intrinsic network structure of a subarea. In general, considering the 2^*nd*^ to the 5^*th*^ order is fine. The point-wise motifs can indicate a subarea’s PT performance with an accuracy of about 60–70% when $t=2$ (Table 1). Given that the measure only takes into account the geometric and topological structures of the PT network, the accuracy is acceptable. The finding justifies that even without considering much observational data we can pre-evaluate a designed PT system with some level of confidence.

**Table 1:** Graph classification accuracy**.

Convolution times				Output
1	2	3	4	
0.626	0.699	0.593	0.652	${l}_{\tilde{t}}$
0.622	0.646	0.687	0.498	${l}_{\tilde{d}}$

**With a variance of ±0.170.

#### Indicating PT system performance

We have seen that the proposed motifs can indicate the PT efficiency and equality. Here the spatial contexts and characteristics for different types of PT performance are further investigated. Ideal subareas have both high efficiency and high equality (orange circles) of PT travel. This category infers that different O/D pairs in the subarea equally have a short travel time. Subareas located at the inner circle of Beijing normally belong to this category (Fig. 2A). These subareas are densely and evenly embedded with stops of high-mobility motifs, e.g., transfer stops and subway stations. High equality no longer holds true if the PT network and stops are not evenly distributed. In that case the spatial distribution of different mobility motifs demonstrates unevenness. In Fig. 2B, there is only one subway station in the middle of the subarea. Only the residents near the subway station are more connected with the rest part of the city. Local travels within the subarea cannot rely on subway, neither on bus because of its low density. By the auxiliary information from satellite images and the house-rental websites, we identify quite a few recently built residential areas that are located in the low-mobility areas. The local residents have to rely on cars if no more PT infrastructure is to be constructed; otherwise the residents are constraint by limited resources.

High equality does not necessarily mean high efficiency. Figure 2C is a subarea where the whole range has “equally” poor access to PT services. The shortage of high-mobility motifs effectively red-flags the low PT efficiency in this subarea, where special attention from planning and policies should be drawn. While suffering from a vacancy of basic facilities, a majority of big residential areas and schools in this range, including “Cui Cheng” and “Lv Zhou Jia Yuan” (marked as “CC” and “LZ” on Fig. 2C), are not covered by PT network unless after a 20 minutes’ walk. The convenience of daily basic needs such as shopping in a supermarket cannot be guaranteed.

One incapability of the current measure and motifs is to capture the influence of road network shape. PT efficiency is decided by the relative ratio of PT travel time/distance to the corresponding shortest network distance ($\tilde{t}$, $\tilde{d}$), for which reason a sparse network yields a higher efficiency. The motifs, since being localized for a small context (up to *t*-order neighbours) around an ego node, cannot coalesce the road network shape of a bigger range. A typical example is shown by Fig. 2D, where the subarea with a big national park and heterogeneous landscape. Our current measure (Eq. 1) does not yet incorporate the network properties of a more global scale, e.g., the betweenness centrality. It potentially can be combined into the unified formula as one dimension of edge weight. The last case is an outlier (Fig. 2E) where the PT network seems dense but the travel efficiency is not good as. The reason might be attributed to the viaduct that induces detour, and the airport express (going northeast-southwest direction) that do not take the role of a normal subway line.

## Discussions

In alignment with Mishra *et al*.^16^ to propose less observation-dependent measures, we come to graph theory and go beyond the conventional centrality measures. Existing centrality measures, e.g., SNAMUTS (http://www.snamuts.com/nodal-connectivity1.html) and Mishra *et al*.'s, only represent the connectivity of the *ego* node instead of a locality, while the proposed measure takes into account the centralities of not only the ego node but also its neighbourhood up to the *t*^*th*^ degree. The convolution-inspired measure as well as the corresponding motif captures the distribution, combination, and connection of the neighbour nodes with different centralities, and aggregates them to the ego node. Our experiment validates the significance of morphology in deciding PT performance; the method demonstrates spatial heterogeneity of motifs in PT multigraph and thus explains why PT performance varies from place to place. When an ample dataset is not available or not necessary, the proposed motifs as a deputy of network morphology can approximate PT performance to some extent. Moreover, the convolution-inspired measure is unified and extensible to incorporate multiple factors under concern and thus provides an integrated encoding. Node, line, and transfer properties are all built in the same formula (Eq. (1)) as a point-based measure containing not only the node and edge properties but also the whole surrounding connectivity. Transfer information is reflected by node properties. More factors of choice, such as PT service time table, travel demand, and lane capacity can be incorporated. For example, Blanchard and Waddell’s UrbanAccess^25^ considers operation schedules of lines on a multi-modal PT network. Our measure can be adapted to incorporate dynamic constraints by introducing a time-dependent multiplier to link weight (see Methods, Eq. (3)). The size of the surrounding to be considered for connectivity is also adjustable, so is the number of extracted motifs.

Methodologically this paper suggests a solution for graph classification with continuous numerical edge weights, when motifs are unknown ahead of time and meanwhile enumeration of motifs is impossible. This is a crucial contribution to dealing with combinatorial complexity and infinity of subgraphs that are confronted by the conventional methods to solve this problem. The gist of our method is to convert the subgraph property and structure to a point-based vector, based on which motifs are drawn. The method is not limited to PT multigraph analysis but can be extended to deal with all types of graphs with continuous numerical weights or unknown motifs, for example, the mobile phone call network^26,27^, the movement flow network^28,29^, and street networks^23^.

Future works may consider the following directions. This method is not for route planning as there is no route specific indicator. However, travel efficiency varies from route to route. A route-based indicator that takes into account travel demand dynamics thus is of interest. We also aim to conduct multiple cities’ case studies to understand the variety of cities stemmed from PT network morphology and their different attractiveness. Another topic is to investigate the scale of motifs in geographic networks, since the modifiable areal unit problem may emerge and some studies outside geography (e.g., Knabe *et al*.^30^) found non-localized entanglement between motifs.

## Methods

**Figure 3:**
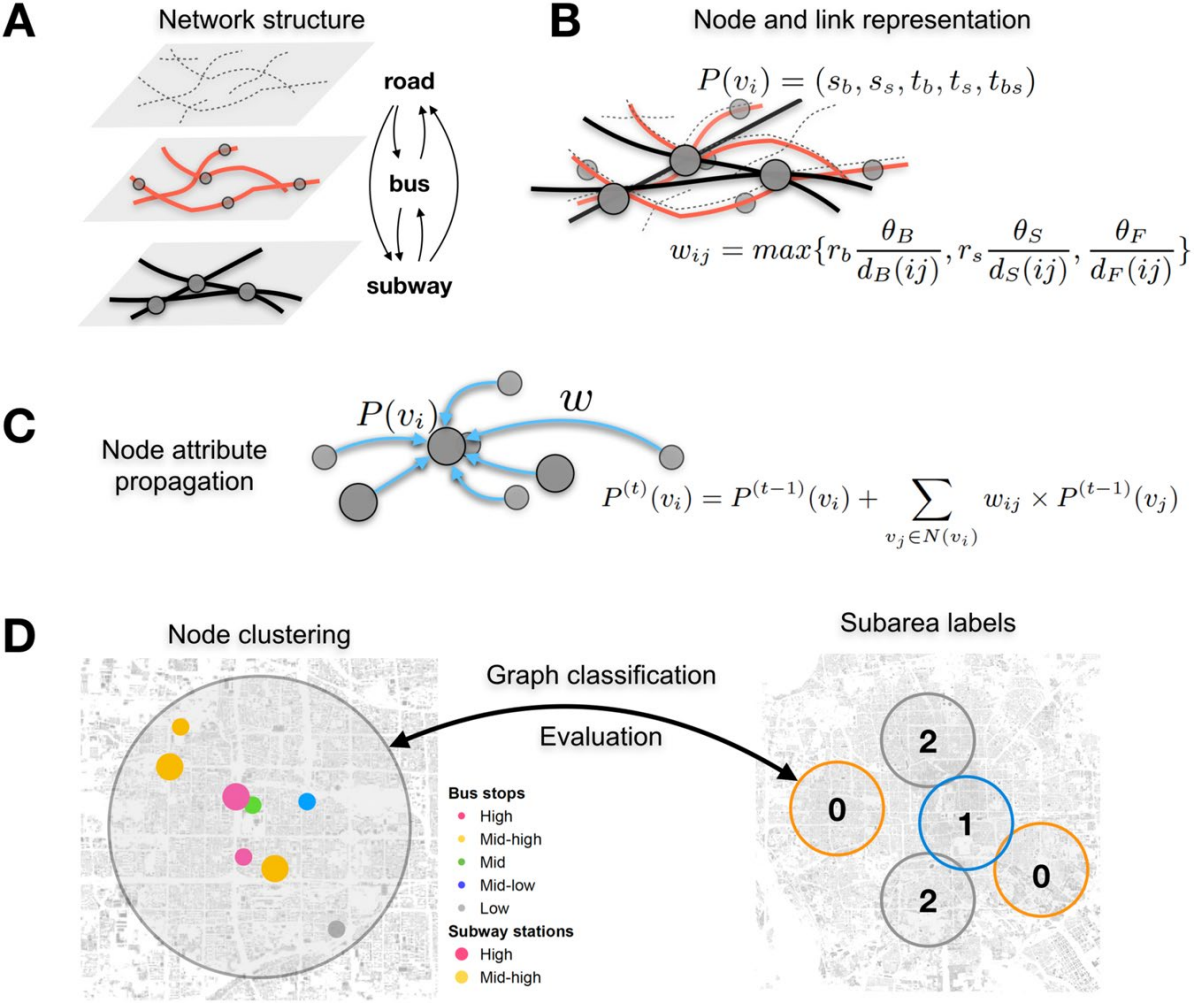
Assessing the node-based performance of PT spatial multigraph: the workflow. (**A**) Building the spatial PT multigraph from subway, bus, and road networks. (**B**) Node and link representation. Each node has a 5-dimensional vector representation. Each edge is weighted by considering the travel speed, mode, and distance. (**C**) Propagating node properties from the local neighbourhood to the ego node as in convolution. (**D**) Using node clusters to predict subarea PT performance classes as validation of the measure. The left graph (a zoom-in to one subarea) is the input of the classification, i.e., clustering nodes into performance levels based on the output representation (motifs) after convolutions. The right hand graph is the output of classification, i.e., the labels of each area based on its adjusted travel time and distance.

The proposed assessment of PT travel efficiency and equality – together called *PT performance* – takes four steps as illustrated by Fig. 3: A. constructing the spatial multigraph from public transport layers; B. representing each node with a feature and calculating edge weights; C. propagating and aggregating the neighbour nodes’ properties via their connectivity with the ego node; D. with the output node feature from C, clustering the nodes into categories as point-based travel convenience measures, which are applied to indicate the travel performance of the corresponding region (circle). Step A is the foundation of the analysis, Steps B and C depict the essential method, and Step D is the evaluation of the proposed method.

### Node and edge representation

A node ${v}_{i}\in V$ has an initial attached 5-dimensional vector $P({v}_{i})={P}^{\mathrm{(0)}}({v}_{i})$ (Eq. (2)) to represent the type of the node, i.e., whether a node is a bus stop, a subway station, a bus transfer stop, a subway transfer station, or a bus-subway transfer stop (where walking between bus and subway is shorter than 100 m). If a node is none of the above, then it is an origin/destination (O/D) Fig. 3A,B. The initial nodal vector ${P}^{\mathrm{(0)}}({v}_{i})$ is binary.

2 $$P({v}_{i})=({s}_{b},{s}_{s},{t}_{b},{t}_{s},{t}_{bs})$$

Links exist not only between the same type of network nodes, but also between different types of nodes considering transfer links. The latter is called *cross-layer links*, including bus-subway transfers and the first/last mile transfers between an O/D and a bus/subway station. The nodes directly connected to an ego node ${v}_{i}$ by any link in *M* are called the *neighbours* or *alters* of ${v}_{i}$, i.e., $N({v}_{i})=\{{v}_{j}\in V|({v}_{i},{v}_{j})\in E\}$. For implementation, if the link length is shorter than a threshold (e.g., 100 m), we treat both nodes as a bus-subway transfer, i.e., assigning ${t}_{bs}=1$. The first/last mile transfer edges are built up by adding walking paths between ODs and all the stations within 500 m (or the nearest station if no station exists within 500 m).

The weight of a link is a comprehensive measure of the network distance ${d}_{(\cdot )}(ij)$ by a certain mode, the travel mode coefficient ${\theta }_{(\cdot )}$ quantified as a mode’s travel speed, and the number of operating bus/subway lines ${r}_{b}$, ${r}_{s}$ between two stops. We only assume transfers by walk, so there are three modes: by bus ($B$), by subway ($S$), and by walk ($F$). Refer to Supplementary Note 1 for how to calculate ${\theta }_{(\cdot )}$. Equation (3) gives the specific formula of the weight by each type of travel mode. If more than one type of link exists between two nodes, the maximum weight will be reserved. The formula can be adapted to incorporate the line operation constraints (e.g., time of operation) by introducing a binary multiplier ${I}_{ij}(h)\in [0,1]$ of ${w}_{ij}$ depending on the hour $h$ of day.

3 $${w}_{ij}=max\left\{{r}_{b}\frac{{\theta }_{B}}{{d}_{B}(ij)},{r}_{s}\frac{{\theta }_{S}}{{d}_{S}(ij)},\frac{{\theta }_{F}}{{d}_{F}(ij)}\right\}$$

### Convolution and node propagation

Node convolution can be conducted multiple times ($t$) in a recursive manner, of which the output is no longer a binary vector Fig. 3C. Only when $t=0$, we have ${P}^{(0)}({v}_{i})=P({v}_{i})$ as a binary vector. More times of convolutions involve nodes of a bigger neighbourhood. In every one more time of convolution, the nodes of one more degree of connectivity are incorporated into the updated ego node vector, even though explicitly we only consider the representations of directly connected nodes. See Algorithm 1 for details. The *t*-th time of convolution brings the attributes of the $t$-th degree to the ego node vector. There is a distance decay effect of node attributes: closer nodes are calculated more times than further nodes, and thus contribute higher weight. The influence of times of convolutions are discussed in Supplementary Note 5.

#### Algorithm 1 . Node attribute propagation.


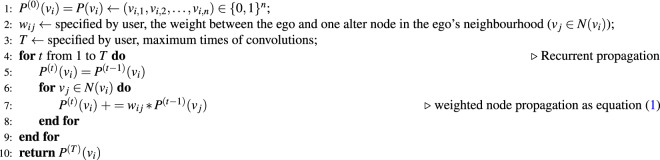


### Graph classification: from motifs to PT performance

Graph classification is applied to project motifs of a PT multigraph to its PT performance Fig. 3D. On the input side we have derived motifs as the substructures that are typical and representative for a category of graph, while on the output side a graph is assigned with a label based on the observed PT performance. Each of the 45 sampled subareas is modelled with a multigraph. Here we show how to calculate the output label for each multigraph according to its PT performance, and how to map the input side motifs to the output side labels.

#### The observed PT performance from real map service

To calculate the target label, we inspect PT efficiencies in different subareas of the city (Fig. 3D). Supplementary Figure 1 demonstrates the workflow to retrieve the empirical PT performance. As discussed above, only short- to medium-distance travels, i.e., within a 4 km diameter circular area, are considered. A further discussion on the influence of buffer size is given in Supplementary Note 5.

We fetch the area within the 5^*th*^ Ring Road of Beijing, where 45 pieces of 4 km-diameter circles delineate the subareas for comparison. The numbers are decided so that sufficient samples are drawn to investigate the variance of efficiencies while avoiding blank or overlap between subareas. In each subarea, the travel efficiency and equality respectively are approximated by the mean (*μ*) and the variance (*δ*) of an adjusted PT travel time ($\tilde{t}$) (or distance ($\tilde{d}$)) of the sampled OD points (Supplementary Fig. 1D,E). We scatter sample points along each road segment with an equal interval of 200 m and pair every each two of them as OD pairs. The PT travel time ($t$) and route length ($d$) between a pair are calculated by querying Amap API (https://lbs.amap.com/api/webservice/summary/). The returned travel time, according to the API provider, is the average travel time at different time of day. Note that we do not apply the General Transit Feed Specification because it is not publicly available in China, and buses do not stick to the schedule for many reasons. $t$ and $d$ are divided by its corresponding shortest walking network distance (${d}_{N}$) to tease out the influence of distance, i.e., $\tilde{t}=t/{d}_{N},\tilde{d}=d/{d}_{N}$. A sampled subarea’s PT performance thus can be quantified by (${\mu }_{\tilde{t}},{\delta }_{\tilde{t}}$) if assessed by time and (${\mu }_{\tilde{d}},{\delta }_{\tilde{d}}$) if by distance. We conducted a k-means clustering of subareas with (${\mu }_{\tilde{t}},{\delta }_{\tilde{t}}$) or (${\mu }_{\tilde{d}},{\delta }_{\tilde{d}}$) to gauge the similarity of their PT performances in time or distance. Labels ${l}_{\tilde{t}}$ and ${l}_{\tilde{d}}$ are the outputs for each subarea to be applied by graph classification.

#### Graph classification

We count the number of each motif ${\tau }_{m}$ in a subarea’s multigraph *M* as *M*’s vector representation (Eq. 4, |*T*| is the total number of motifs in the whole graph *M*). $\phi (M)$ denotes the process of graph classification (Supplementary Fig. 1F). Supporting Vector Classifier is applied for its simplicity and effectiveness. The classification accuracy (Table 1) displays how capable the measure can infer PT efficiency.

4 $$\phi (M)=(\#({\tau }_{1}\subseteq M),\ldots ,\#({\tau }_{|T|}\subseteq M))$$

In the Supplementary Note 7, we also demonstrate the results of indicating PT efficiency by some aggregative level statistics considering multiple factors. The convolution based graph classification generally yields a better result.

## Supplementary information

Supplementary Information.

## Data Availability

We applied the basic road network edited from OpenStreetMap https://www.openstreetmap.org, and clipped to the 5^*th*^ Ring Road with ArcMap. The bus and subway networks of 2017 were bought from a data vendor. We have the dataset available at https://github.com/jikewenqing/PT_efficiency_multigraph/blob/master/Beijing_subway_bus_data.7z.
