# Supplementary material for: A unified spatial multigraph analysis for public transport performance
Source: Sci Rep. 2020 Jun 12;10:9573. doi: 10.1038/s41598-020-65175-x (PMC7293237; doi:10.1038/s41598-020-65175-x)
Supplement: Supplementary file 1 — Supplementary Information. [file 41598_2020_65175_MOESM1_ESM.pdf]

Supplementary Methods: A unified spatial multigraph analysis for sustainable urban transportation

Yaoli Wang[1,2], Di Zhu[1,2], Ganmin Yin[1,2], Zhou huang[1,2,*], and Yu Liu[1,2]

[1]Institute of Remote Sensing and Geographical Information Systems, Peking University, Beijing, 100871, China
[2]Beijing Key Laboratory of Spatial Information Integration and Its Applications, Peking University, Beijing 100871, China
[*]huangzhou@pku.edu.cn

Contents

1 Calculating θ 5

2 Spatial graph complexity 6

3 Ground truth 7

4 Clustering of graphs by travel efficiency 7

5 Influence of scale 9

6 Cluster pattern 9

7 Prediction with aggregative statistics 13

References 16

List of Figures

1 The evaluation of PT performance by subareas and Amap API 7
2 Distributions of $\tilde{t}$ and $\tilde{d}$ of subareas. 8
3 Clustering result of PT efficiencies of different sizes of subareas 11
4 Node clustering with travel time. 12
5 Node clustering with travel distance. 13
6 OD node clustering result after twice convolutions. 14

List of Tables

1 Travel time and distance of different (pure) modes 5
2 Estimation of travel speed 6
3 Graph classification accuracies 10

1 Calculating θ

As shown by Eq. 3 in the main text, the $\theta_{(\cdot)}$ is a key parameter of edge weight. Intrinsically it is the proxy of the average travel speed of a certain mode. Calculating the travel speed of a certain mode is tricky since the waiting time at a stop should be excluded, and the travel speed is heterogeneous over space due to road conditions and configurations. As an approximation to up-to-date routes and travel time, we applied Amap API[1] to query the travel distance and time between selected subway stations. The estimated travel time queried from Amap API is regardless of the traffic situation at different time of day. In order to calculate the travel speed of each pure mode for the same route, walking time and distance to bus stops are deducted from bus travel time and distance.

Table 1. Travel time and distance of different (pure) modes between sampled stations along a selected subway line

Stations (n)	mode	distance (km)	time (min)	avg. speed (km/h)
S_0S_1 (1)	subway	0.9	2	27.00
	bus	0.6	9	4.02
	walk	0.9	13	4.14
S_0S_2 (2)	subway	1.7	4	25.50
	bus	1.2	15	4.80
	walk	1.8	25	4.32
S_0S_3 (3)	subway	2.9	6	28.98
	bus	2.3	20	6.90
	walk	2.9	39	4.44
S_0S_4 (4)	subway	4.0	8	30.00
	bus	3.4	22	9.30
	walk	3.9	53	4.44
S_0S_5 (5)	subway	5.6	11	30.54
	bus	5.1	31	9.90
	walk	5.6	74	4.56

The selected subway stations all have proximity to bus stops. We sampled the stations along a few subway lines. For each line, the selected station pairs all share the same start station S_0, while end at each consecutive station S_n with n ($n = 1,2,3...$) stops away towards one direction. This operation allows us to do differential calculation to tease out the waiting time at a station, e.g., using time of S_0S_n minus S_0S_{n-1} as the travel time of $S_{n-1}S_n$ ($n > 2$). Corresponding travel distance is calculated in the same manner so that we can estimate the travel time. Table 1 displays an example (Subway Line 4) of the sampled travel time and distance to approximate $\theta_{(\cdot)}$, starting from East Gate of Peking University (S_0), going through Zhongguancun (S_1), Huangzhuang (S_2), Renmin University (S_3), Weigongcun (S_4), and ending at National Library (S_5).

We observed that the number of stops on a route drastically affects the estimated travel speed, especially for bus. The overhead costs such as boarding and alighting, walking in a station, are more significant for shorter trips. Buses are more prone to the influence since they take longer time to accelerate and decelerate than subway and normally have closer stops.

Table 2 is the differential result between each entry from the second and the first entry ($S_0S_n - S_0S_1$ ($n > 1$)) in Table 1. This operation removes the overhead cost at the origin stop of each trip. θ is estimated by the average travel speed of each mode. Since only the ratio between different modes matters, we let $\theta_F = 1$. According to Table 2, we let $\theta_B = 2.5$ in spite of its variance in shorter distance. $\theta_S = 6.7$ is also inferred from the table.

As a comparison, we also let $\theta_B = 1$ and $\theta_S = 6.5$, testing the scenario when the travel speed takes into account the overhead cost at each stop, e.g., waiting for boarding or alighting, acceleration and deceleration. The second half of Table 3 shows the result from such setting. This is the lower bound of speed derived from Table 1 where the last column is the averaged speed considering mixed mode as well as multiple overhead cost. Apparently the average travel speed by subway and by bus will be lower for shorter distance, e.g., S_0S_1, than for longer distance. Bus is even slower than walking for S_0S_1 considering the time of waiting. Since Table 2 calculates the travel speed after teasing out the overhead cost of first/last mile walking and waiting, it yields the upper bound ratio of θ_B/θ_F and θ_S/θ_F.

[1] https://lbs.amap.com/api/webservice/summary/

Table 2. Estimation of travel speed of different modes by differential operation

Stations	mode	Δdistance (km)	Δtime (min)	$\bar{v}^{(\Delta n)}$ speed (km/h)
S_1S_2	subway	0.8	2	$\bar{v}_S^{(1)} = 24.0$
	bus	0.6	6	$\bar{v}_B^{(1)} = 6.0$
	walk	0.9	12	$\bar{v}_F^{(1)} = 4.5$
S_1S_3	subway	2.0	4	$\bar{v}_S^{(2)} = 30.0$
	bus	1.7	11	$\bar{v}_B^{(2)} = 9.3$
	walk	2.0	26	$\bar{v}_F^{(2)} = 4.6$
S_1S_4	subway	3.1	6	$\bar{v}_S^{(3)} = 31.0$
	bus	2.8	13	$\bar{v}_B^{(3)} = 12.9$
	walk	3.0	40	$\bar{v}_F^{(3)} = 4.5$
S_1S_5	subway	4.7	9	$\bar{v}_S^{(4)} = 31.6$
	bus	4.9	22	$\bar{v}_B^{(4)} = 12.5$
	walk	4.7	61	$\bar{v}_F^{(4)} = 4.6$

2 The complexity of spatial graph classification

This section justifies why we invented the convolution-based method for graph classification. Combinatorial complexity is a big challenge to compute frequent subgraph patterns in graph boosting[1–4]. Traditional graph boosting requires enumerating appeared subgraph patterns. For a binary 5-dimensional vector the possible statuses of each node is $2^5 = 32$. Due to the dependency between dimensions, only 16 statuses are possible in the case study. If a stop is none of the specified transport stops/stations, it cannot be a transfer stop/station either (Eq. 2 satisfies $t_b \leq s_b, t_s \leq s_s$); and a node has to be either a bus stop or a subway station ($s_s + s_b \geq 1$ in Eq. 2) since we do not consider nodes with only the role of road intersection. Let the degree of a node be d, thus alter nodes' combinatorial statuses sum up to 16^d. If linkage status is discrete with 3 possible statuses, e.g., the three modes as bus, subway, and walk, then the 1-degree connections of a node jointly yields 3^d combinations. In total $(16^{d+1})(3^d)$ $(d \in N^+)$ potential subgraphs emerge for only one node's 1-degree neighbourhood.

In our case, it is more complicated since the link weight can have numerical continuous values or the possibilities of link weights are from an unpredictable infinite set. It is very challenging in that sense to clearly define a subgraph. Geography has the challenge of high complexity in human society. We attributed the complexity of analysing a spatial network to the following points:

- Spatial network is mostly a *multigraph* in which multiple types of links (i.e., interactions between places such as traffic flow, social network connection, etc.[5]) can simultaneously exist. Solving the tensor adds complexity to solving a matrix. For example, place A and place B can at the same time have interactions of traffic flow, bank transactions, and social network connections. Treating the networks independently and separately is not ideal if the research purpose is to find the association across different layers of networks.
- The topological structure of a spatial network can appear so complex that enumeration of motifs are unlikely to conduct. The comparison of spatial networks in different places as well as scales is challenging to perform.
- Applying neural network based method for spatial networks is more challenging than for the networks in fields such as biomedical sciences or online digital behaviours due to an equal (if not higher) level of complexity while higher difficulty in collecting observation data of real-life scale.

We are hence inspired by the capability of convolution to handle continuous edge weight, but avoided the parameter training mechanism due to the very small amount of observations. This is also the base of argument for Section 1 to choose a suitable θ.

3 The illustraton of calculating the observed PT travel time and distance as ground truth

Fig.1 shows the workflow to retrieve the PT travel time and distance as ground truth. We called Amap API for this analysis instead of using the bus timetable because bus schedules are not accurately released in China and buses mostly are not running on schedule either. We argue that calling Amap API is generally more reliable.

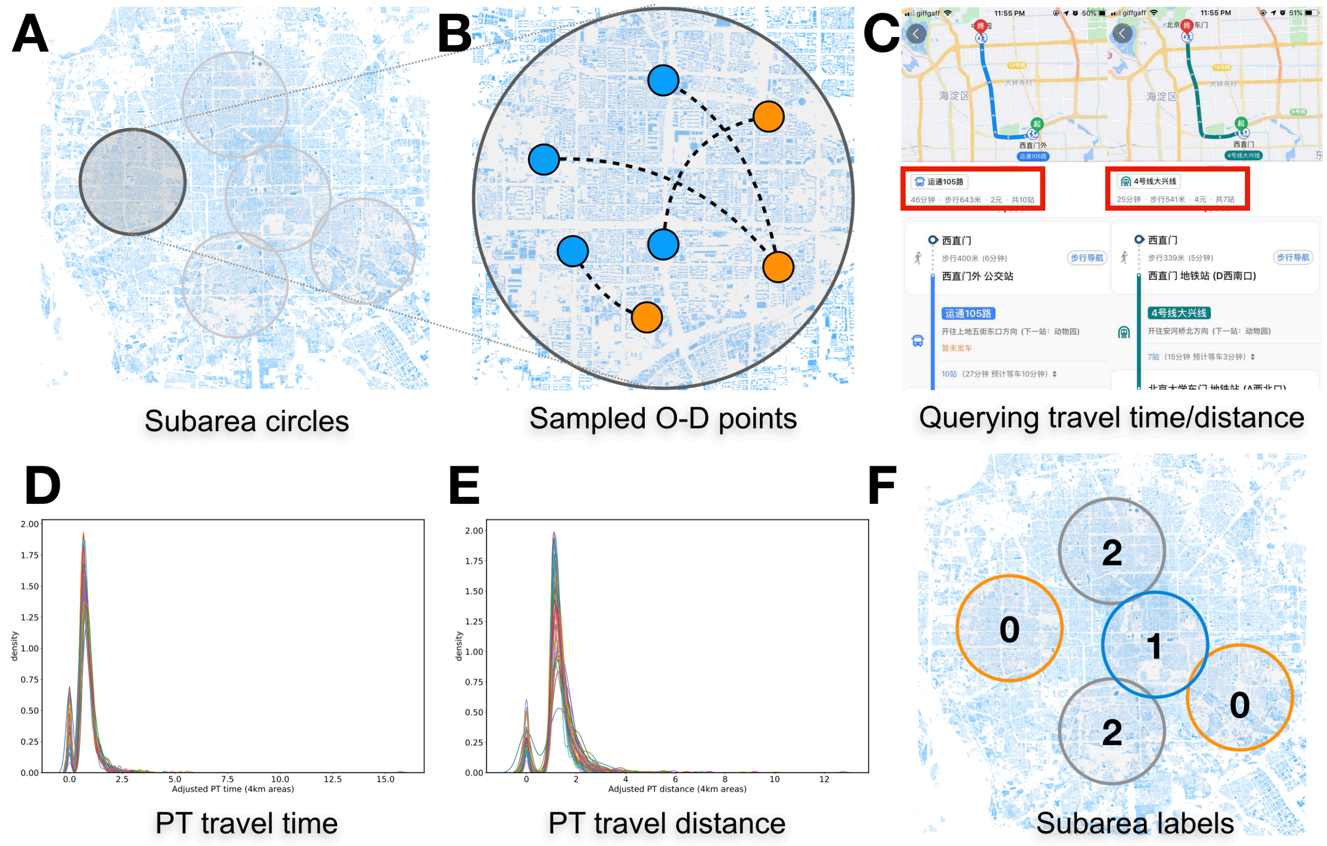

Figure 1. The evaluation of PT performance by subareas and Amap API. (A) Sampling subareas in the city with 4km diameter buffers. (B) Retrieving sampled O/D points by each subarea. (C) Querying with Amap API the PT travel time and distance between each pair of O/D points. (D) The distribution of PT travel time. One curve corresponds to one subarea. Derived are the mean and variance of the travel time $(\mu_{\tilde{t}}, \sigma_{\tilde{t}})$. (E) The distribution of PT travel distance. One curve represents one subarea. Derived are $(\mu_{\tilde{d}}, \sigma_{\tilde{d}})$. (F) By clustering the subareas based on $(\mu_{\tilde{t}}, \sigma_{\tilde{t}})$ or $(\mu_{\tilde{d}}, \sigma_{\tilde{d}})$, subareas are labeled with the cluster numbers $l_{\tilde{t}}$ or $l_{\tilde{d}}$ as the deputy of PT performance. Circles on this subfigure are not to scale.

4 Clustering of graphs by travel efficiency

Clustering of graphs by travel efficiency categorizes graphs based on their *observed* performance. Labels resulted from this clustering are for the output side of graph classification in the main text ("subarea labels" in Fig.3D). Here we demonstrate the results for three sizes of areas, 3km, 4km, and 5km diameter areas. In the main text we only discussed the 4km area cases. The travel efficiencies in each subarea – $\tilde{t}$ and $\tilde{d}$ – have a similar distribution to a log-normal distribution at the tail part, but with a minor peak to the left where x = 0 (Fig 2). Note that due to kernel density estimation in visualization, the curves look like extending into negative values but in fact do not. Each line corresponds to a subarea. We did not perform a log-normal transformation because we noticed the heavy tail outliers are a distinguishable character of a subarea. Subareas are clustered by its $(\mu_{\tilde{t}}, \sigma_{\tilde{t}})$ or $(\mu_{\tilde{d}}, \sigma_{\tilde{d}})$ with Supporting Vector Classifier (SVC). We selected SVC because it is an efficient method that is able to handle non-linear classification and works pretty well for a small number of samples (in our case, subareas). The number of targeted classes are specified as three not only to guarantee a high Calinski-Harabaz score (52.89 for $\tilde{t}$ and 110.60 for $\tilde{d}$) – the score to measure the goodness of clustering when the real cluster label is missing – but also to yield a small amount of clusters for the easiness of human interpretation.

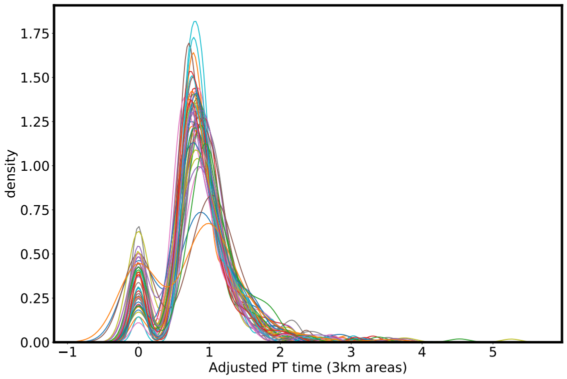

(a) Distribution of $\tilde{t}$ of 3km areas

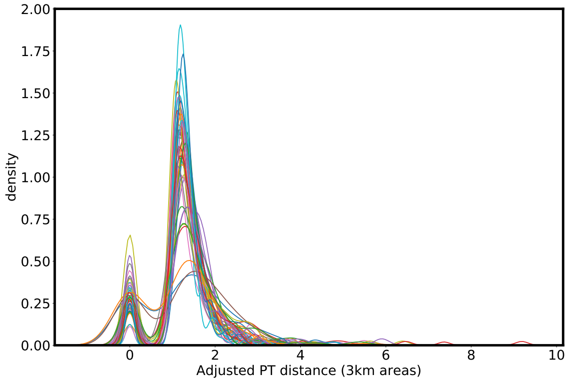

(b) Distribution of $\tilde{d}$ of 3km areas

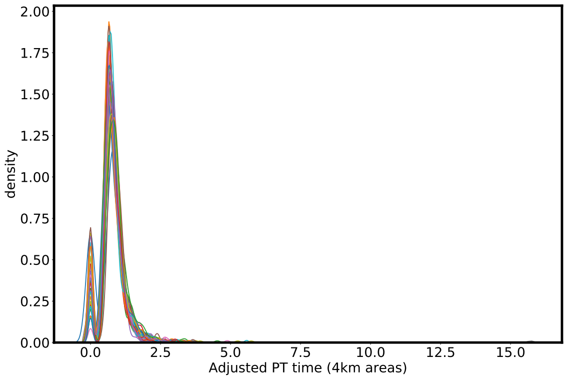

(c) Distribution of $\tilde{t}$ of 4km areas

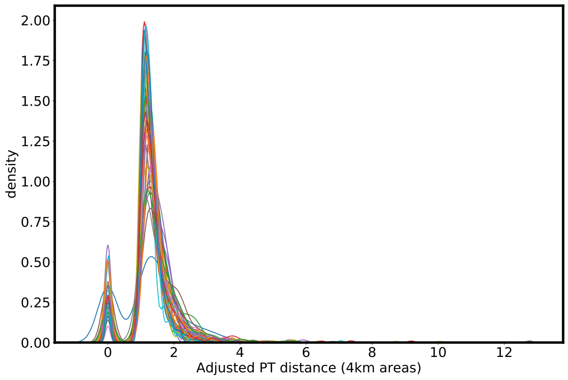

(d) Distribution of $\tilde{d}$ of 4km areas

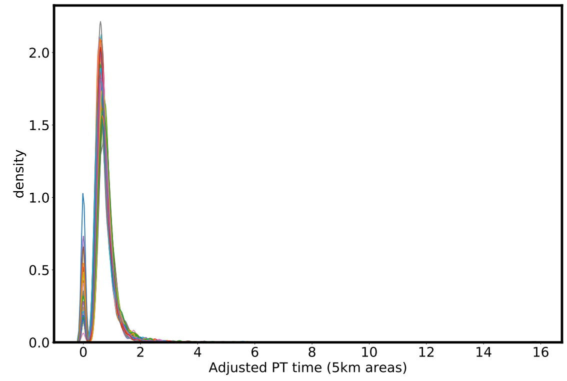

(e) Distribution of $\tilde{t}$ of 5km areas

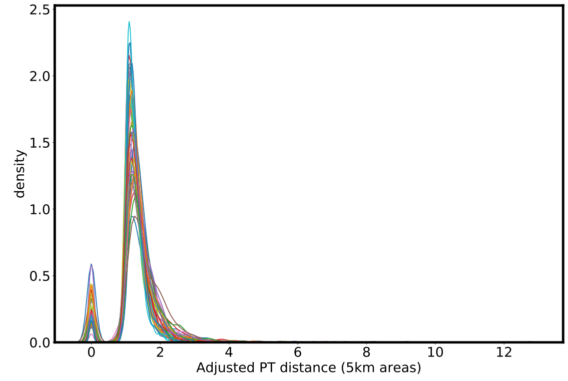

(f) Distribution of $\tilde{d}$ of 5km areas

Figure 2. Distributions of $\tilde{t}$ and $\tilde{d}$ of subareas. Each curve corresponds to one subarea's distribution of adjusted PT travel time or adjusted PT travel distance. The distribution is sampled from the OD pairs falling inside a subarea of 3km, 4km, or 5km diameter.

5 The influence of subarea size and times of convolution

We noticed the difference in the prediction results for different buffer sizes. Table 3 displays the prediction accuracies from graph classifications by the buffers of 3km, 4km, and 5km diameters (by conducting the operations in Section 4). The upper half is yielded by the default setting with $\theta_S = 6.7$, $\theta_B = 2.5$, $\theta_F = 1$, while the lower half is from the setting of lower bound θ as explained at the end of Section 1.

The influence of subarea size The best prediction happens mostly at 4km diameter. We think 4km can evenly cover the whole study area while without too much overlap. The 3km areas in general display the lest capability of prediction. The reasons behind may be attributed to multiple factors. Within 3km areas, travels short as 1km or less may not worth taking public transit at all given the overhead cost of waiting and detour. The queried results from Amap API for the very short travels thus either involve a bigger portion of walking trips even we queried "by public transit", or yield no feasible solution by public transit since walking will be faster. For 5km diameter areas, the significant overlap between subareas makes the classification difficult.

The prediction capability differs between the adjusted PT travel time and PT travel distance. Reading the result from Table 3, for 3km and 5km areas, the prediction on travel distance is better than for travel time, while for 4km areas they relatively make a tie. Travel time is more difficult to predict due to the heterogeneity of operational frequency of public transit services, the road congestion situation, and the unknown overhead cost induced by times of transfers. These factors cannot be modelled by the convolution based method. On contrast, 4km and 5km areas generally can predict PT distance better than applying the benchmark aggregative statistics method (specified in Section 7). The results from 3 times of convolutions to 4 times of convolutions yield a big jump or drop at all scales for PT distance. The reasons behind are not yet clear and are open to further exploration. The sensitivity of the parameters to the results need a systematic test in order to gain clear insights.

The influence of times of convolutions. We also conclude that twice convolutions are already sufficient to represent PT travel time and distance for short distance travels. From the upper half of Table 3 we see that the peak accuracies vary by buffer sizes and by clustering measures (time vs. distance). However, the general trend is that peak values all come no more than 5 times of convolutions. Having more times of convolutions involves the connectivity of larger neighbourhood. Nevertheless, the more degrees of connections a node from an ego node, the more diluted its influence in the convolution process. Compare twice convolutions with five convolutions, the results are not significantly distinguished from each other. We justify the finding by such an intuitive explanation: by research design, setting out from an OD point, the first lag of connections are covered by walking, and the second lag by PT. Intuitively the total travel time is heavily affected by the travel mode of the second lag, because the speed of the mode plays a big role. For instance, if the OD is near a subway station, the second lag of travel is by subway; while if the OD is near a bus stop, then the second lag of travel is by bus. In our experiment setting, travelling by bus is likely to be slower than by subway since the travels are of short distance and thus we usually do not need to consider as many transfers as for long distance travels (transfer leads to overhead cost). If the ego node is a bus stop or a subway station, then the first lag of travel will be of the corresponding PT mode rather than walking. In this case, the connected second degree node depicts more of the convenience of transfers and connectivities. In such a short distance, the load of information by twice convolutions is sufficient.

It is noteworthy that the lower half of the table, i.e., when the travel speed of PT has lower speed, demonstrates a delay of the highest accuracy compared with the upper half of the table. PT efficiency is a comprehensive result of local connectivity, travel speed, travel distance, operational frequency, and road traffic situation. Excluding the last two, which fall out of the capability of the convolution based index, the rest three play a coalesced role. Since bus has no speed advantage over walking in this scenario, more times of convolutions improve the PT efficiency by harnessing the benefit of local connectivity.

We would also like to point out a negative observation – the tendency of homogeneity in the result when the times of convolutions is very high. We tested 20 times of convolutions to find that the node measures converged to the same cluster over the whole graph. In that case, the local neighbourhood of nodes that are involved in node propagation is so wide that the graph is smoothed out. We hence argue that the size of the neighbourhood, i.e., controlled by the times of convolutions, should be capped at a limit. Too many times are not favourable.

6 The spatial pattern of PT performance indicators

Fig. 3 shows the subarea clustering result by different PT efficiencies - $\tilde{t}$ and $\tilde{d}$, where each node has two dimensions μ (mean) and σ (variance). Correspondingly, the overall spatial distribution of PT travel time, i.e., subarea clusters by $\tilde{t}$, of the 4km subareas is demonstrated by the colorful circles in Fig. 4. We did not show the spatial distribution patterns of other subarea sizes nor for travel distance since we focused on the travel time of 4km areas in the main text. The similar method can be applied for the omitted cases. The influence of different buffer sizes is discussed in Section 5. Looking at Fig. 4 and Fig. 3(c)

Table 3. Graph classification accuracies**

$\theta_S = 6.7$, $\theta_B = 2.5$, $\theta_F = 1$										
Diameter	Convolution times								Clustered by	By agg. stats
	1	2	3	4	5	6	7	8		
3km	0.464	0.435	0.394	0.529	0.583	0.552	0.519	0.408	$l_{\tilde{t}}$	0.427
	0.497	0.540	0.470	0.552	0.482	0.464	0.560	0.472	$l_{\tilde{d}}$	0.404
4km	0.626	0.699	0.593	0.652	0.730	0.630	0.605	0.640	$l_{\tilde{t}}$	0.752
	0.622	0.646	0.687	0.498	0.592	0.617	0.605	0.622	$l_{\tilde{d}}$	0.503
5km	0.482	0.528	0.438	0.486	0.458	0.487	0.459	0.456	$l_{\tilde{t}}$	0.583
	0.604	0.649	0.637	0.702	0.657	0.682	0.606	0.589	$l_{\tilde{d}}$	0.549

$\theta_S = 6.5$, $\theta_B = 1$, $\theta_F = 1$										
Diameter	Convolution times								Clustered by	By agg. stats
	1	2	3	4	5	6	7	8		
3km	0.463	0.477	0.441	0.414	0.546	0.684	0.626	0.560	$l_{\tilde{t}}$	0.427
	0.480	0.527	0.484	0.488	0.504	0.486	0.441	0.518	$l_{\tilde{d}}$	0.404
4km	0.628	0.643	0.658	0.569	0.710	0.701	0.652	0.729	$l_{\tilde{t}}$	0.752
	0.622	0.648	0.631	0.664	0.659	0.596	0.646	0.633	$l_{\tilde{d}}$	0.503
5km	0.482	0.474	0.555	0.551	0.556	0.584	0.577	0.559	$l_{\tilde{t}}$	0.583
	0.606	0.623	0.660	0.566	0.670	0.669	0.665	0.662	$l_{\tilde{d}}$	0.549

**With a variance of $\pm 0.160 \sim \pm 0.18$

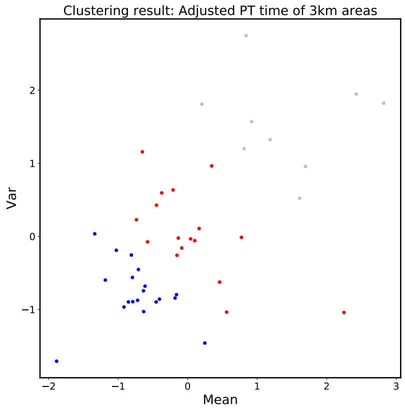

(a) $l_{\tilde{t}}$, 3km areas

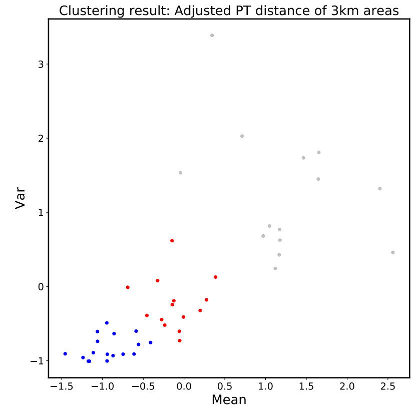

(b) $l_{\tilde{d}}$, 3km areas

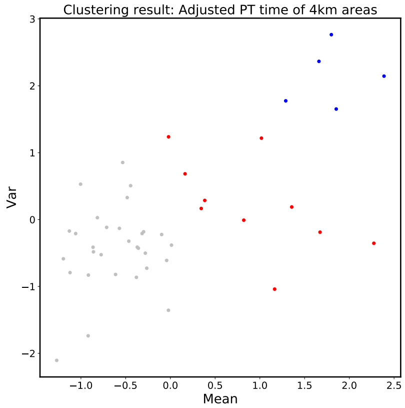

(c) $l_{\tilde{t}}$, 4km areas (gray, red, blue correspond to orange, gray, steelblue in Fig.4 and main text Fig.2)

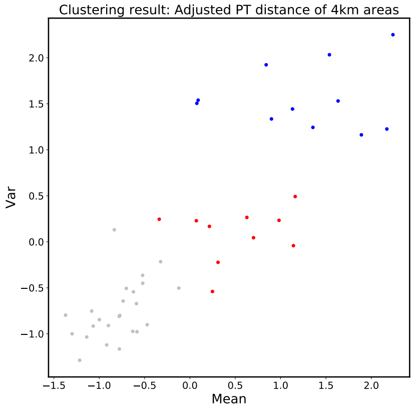

(d) $l_{\tilde{d}}$, 4km areas (gray, red, blue correspond to orange, gray, steelblue in Fig.5.)

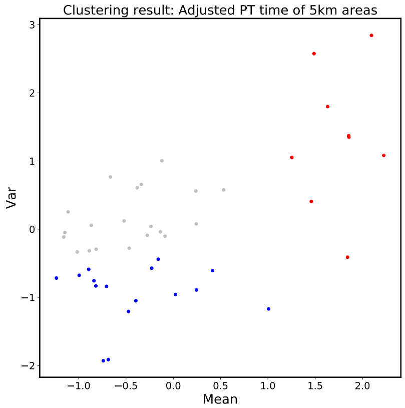

(e) $l_{\tilde{t}}$, 5km areas

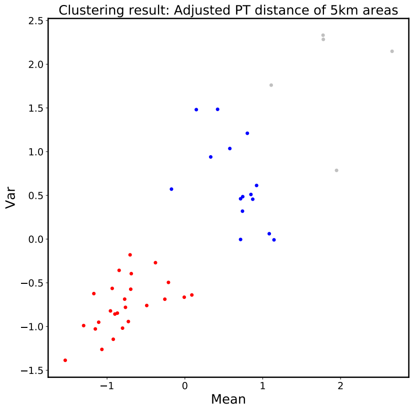

(f) $l_{\tilde{d}}$, 5km areas

Figure 3. Clustering result of PT efficiencies of different sizes of subareas. Colors are randomly assigned and nominally represent different clusters.

side by side, we can see the whole distribution of the most accessible areas (coloured in orange), the less accessible areas with bigger variance of travel time (coloured in blue), and the less accessible areas but with lower variance (coloured in grey).

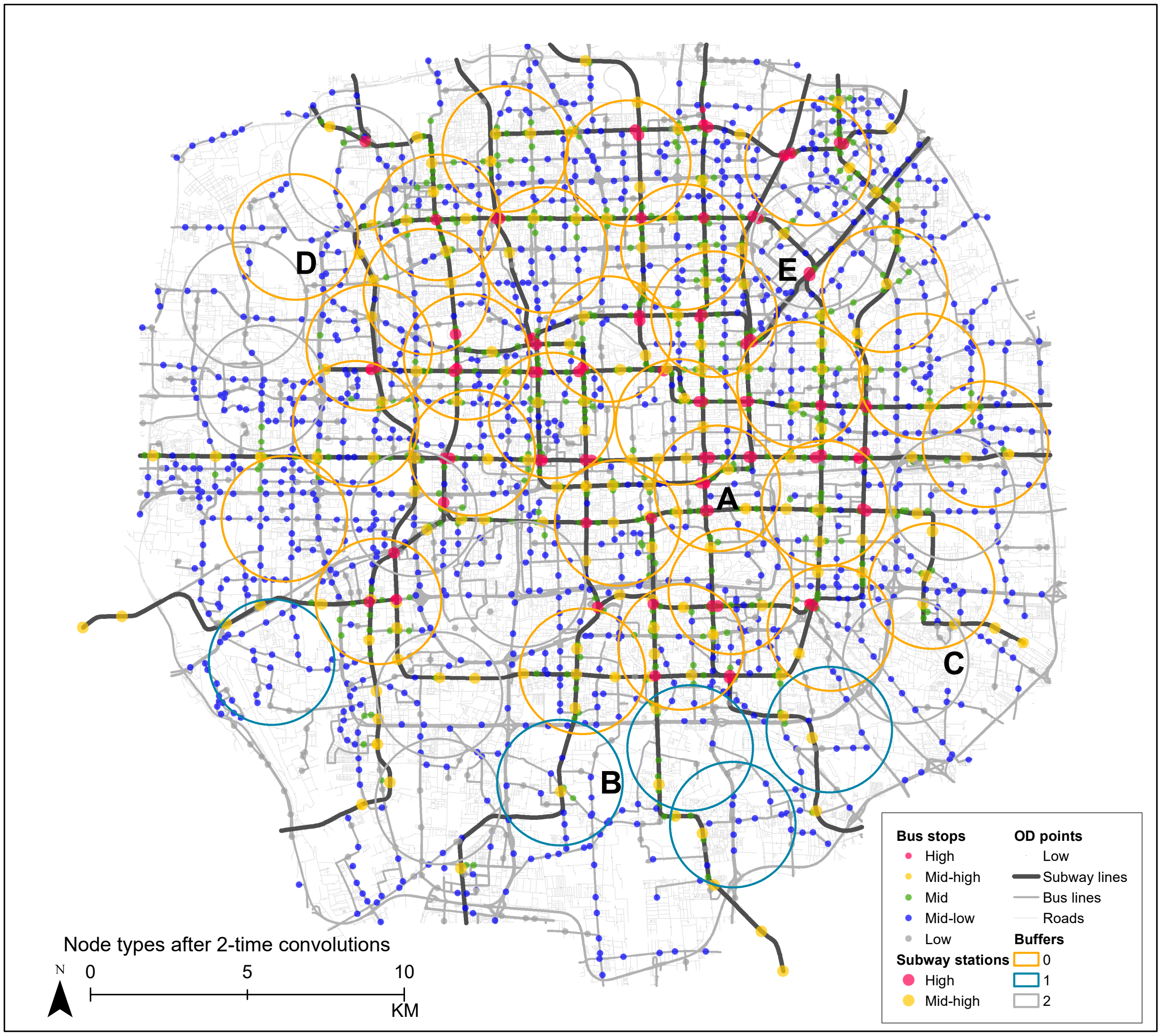

Figure 4. All node clustering result after twice convolutions with the distribution of adjusted travel time. The color of the subarea circles represent the PT efficiency of travel time $\tilde{t}$, as discussed in Section 6.

Normally the most accessible areas are distributed in the central and northern parts of the city, whereas the less accessible ones are located at the southern part and some peripheral areas. This matches the fact that the south edge of Beijing is less developed in terms of commercial and residents' daily activities. The south part, where there are a few warehouses, long-distance shuttle bus stations, and railway stations, takes the function of logistics and warehouse. There are also a few industrial parks over there. The most accessible areas normally have an evenly distributed PT network, including subway lines, sufficient bus lines, and dense road networks. The "evenly distributed" is said with regard to the road distribution. Since we calculated the PT efficiency by normalizing with the shortest network distance, the influence of road network density will affect its PT efficiency. As the main text points out, the top left corner has an orange circle area (as D in Fig.2) that is clustered as high mobility. The area has no subway line at all, but there is a big area of parks and natural reserves that has no roads either. As a result, the bus stops are distributed densely along roads and thus the overall mobility is high.

We clustered the PT efficiency from two dimensions: mean and variance – $(\mu_{\tilde{t}}, \sigma_{\tilde{t}})$ if travel time or $(\mu_{\tilde{d}}, \sigma_{\tilde{d}})$ if travel distance. High variance means the fairness of mobility is low, i.e., the points do not provide relatively equal mobility. With travel time of 4km diameter as an example, in Fig.4, blue-circle areas have some convenient lines going through, e.g., subway lines, while

other parts less facilitated with PT even though the roads are dense (thus the demands are dense in space). However, a high variance does not tell whether the area in general is of higher or lower mobility. When an area is very sparsely facilitated with PT lines (e.g., gray circles), the fairness can be high, i.e., no one has good accessibility to PT. Whereas when and area is densely distributed with PT lines (e.g., orange circles), the fairness is high as well, and all with good accessibility. We omitted the discussion for travel distance but present the spatial distribution of travel distance in Fig.5.

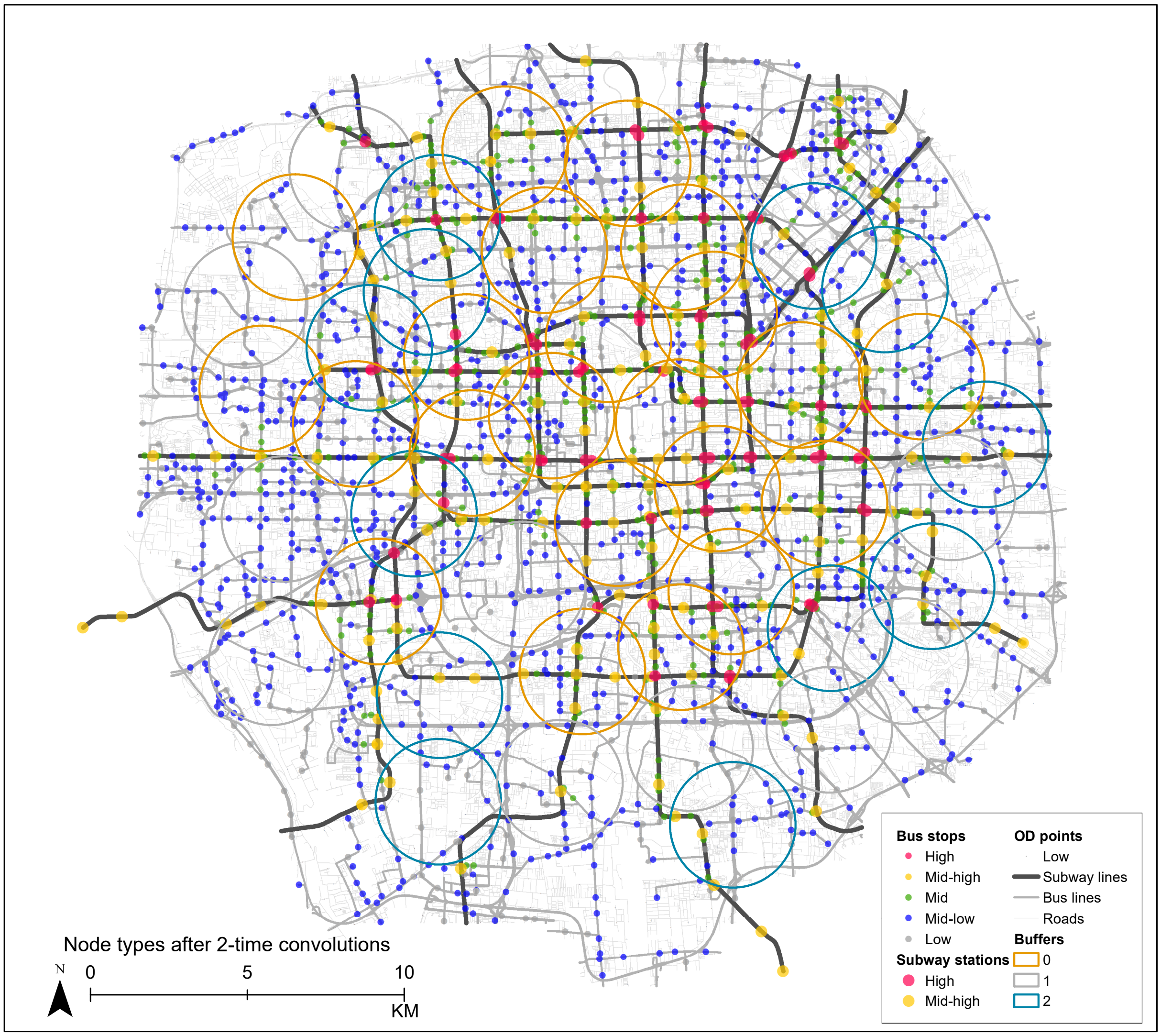

Figure 5. All node clustering result after twice convolutions with the distribution of adjusted travel distance. The color of the subarea circles represent the PT efficiency of travel distance $\tilde{d}$

Road sample points (ODs) are dominated by subway stations and bus stops given the same clustering standard, so we cannot see the difference among the sample points in their accessibilities. To scrutinize more details, we particularly clustered only OD points, yielding the accessibility pattern (see Fig. 6). Discernibly, red OD points indicate the advantage of proximity to subway stations on foot. Yellow ones are a bit far off. Whereas most of the road sample points are in gray, which are only assessed with low accessibility since they are off the stations/stops and only walking is feasible to cover that first/last mile.

7 Predict PT Efficiency with Aggregative Statistics

We compared our multigraph based method with the traditional benchmark index-based method in their capabilities of predicting PT efficiency. We calculated 8 aggregative level statistics for each subarea which were built into an 8-dimension

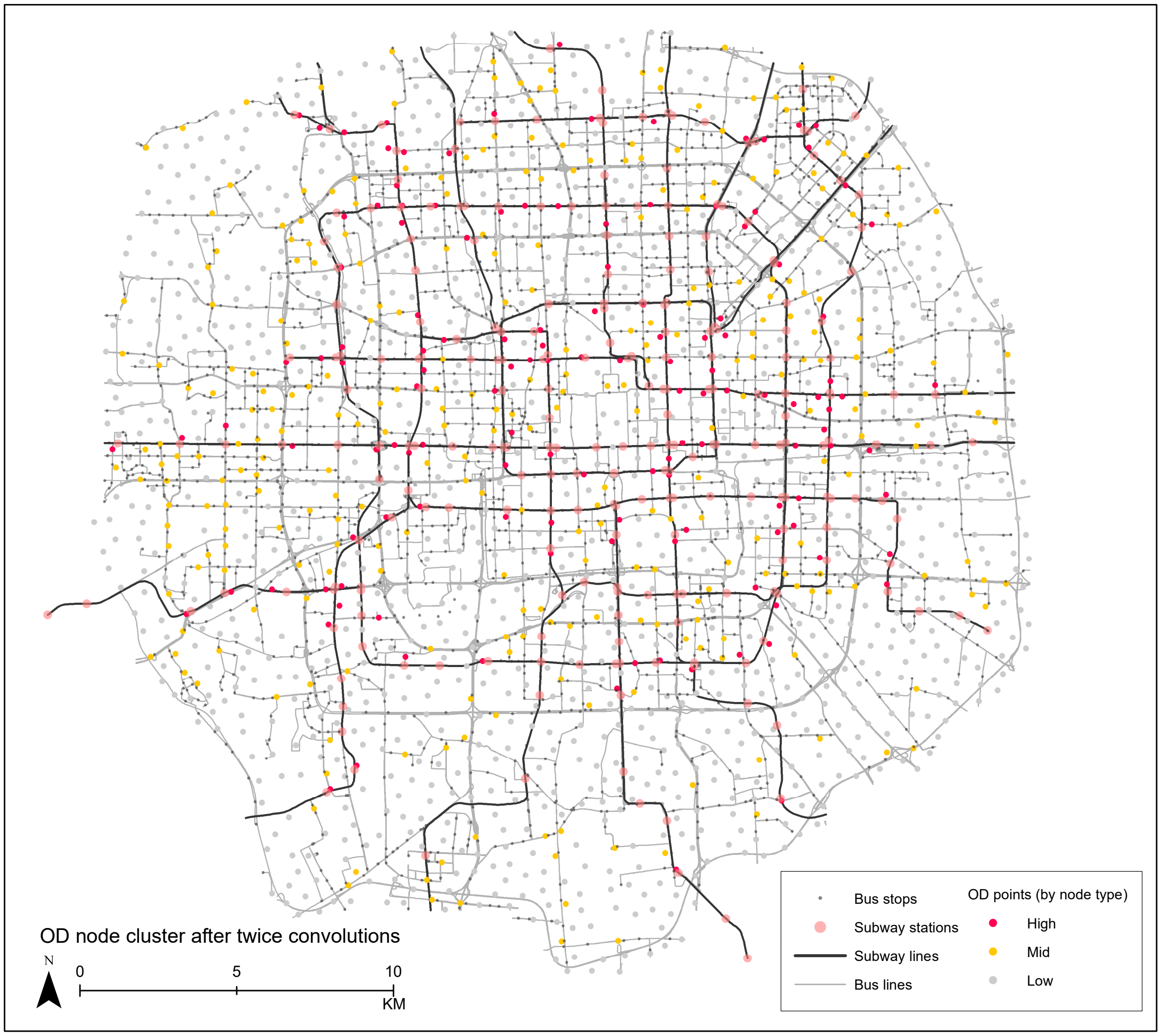

Figure 6. OD node clustering result after twice convolutions.

vector representation for one subarea. The 8 variables include the average lengths of bus routes and subway lines, the numbers of bus stops and subway stations, the average distances to the nearest bus stop and the nearest subway station, the average POI density on each road segment, and the average total road length over the area. The vector was utilized to predict labels $l_{\tilde{t}}$ and $l_{\tilde{d}}$ respectively. The last column of Table 3 shows the result. For predicting PT travel time, the aggregative statistics are slightly but not significantly higher than the convolution based index, while for predicting PT distance, the convolution based index wins over.

References

1. Kudo, T., Maeda, E. & Matsumoto, Y. An application of boosting to graph classification. In *Proceedings of the 18th Annual Conference on Neural Information Processing Systems (NIPS 2004)*, 729–736 (2004).

2. Saigo, H., Nowozin, S., Kadowaki, T., Kudo, T. & Tsuda, K. gBoost: A mathematical programming approach to graph classification and regression. Machine Learning **75**, 69–89, DOI: 10.1007/s10994-008-5089-z (2009).

3. Tsuda, K. & Saigo, H. Graph classification. In *Managing and Mining Graph Data, Advances in Database Systems*, 337–363, DOI: 10.1007/978-1-4419-6045-0 (2010).

4. Wang, H. *et al.* Incremental subgraph feature selection for graph classification. IEEE Transactions on Knowledge and Data Engineering **29**, 128–142, DOI: 10.1109/TKDE.2016.2616305 (2017).

5. Liu, Y. *et al.* Social sensing: A new approach to understanding our socioeconomic environments. Annals of the Association of American Geographers **105**, 512–530 (2015).
